# Psychological and behavioral impact of the COVID-19 pandemic and containment among the general tunisian population

**DOI:** 10.1192/j.eurpsy.2021.747

**Published:** 2021-08-13

**Authors:** D. Gdoura, F. Charfeddine, G. Ktari, A. Chamseddine, O. Bouattour, J. Aloulou

**Affiliations:** 1 Psychiatry (b), Hedi Chaker University hospital, sfax, Tunisia; 2 Psychiatry, Hedi Chaker University hospital, sfax, Tunisia; 3 Psychiatry (b), Psychiatry (B), Hedi Chaker University hospital, sfax, Tunisia

**Keywords:** General population, behavioral impact, psychological impact, covid 19 pandemic

## Abstract

**Introduction:**

Since COVID19 pandemic emergence, containment measure have been taken by the Tunisian government imposing a new lifestyle resulting in psychological repercussions and a change in behavior.

**Objectives:**

Highlighting changes of behavior and lifestyle of the general public secondary to the COVID19 pandemic and to confinement and to assess their levels of psychological.

**Methods:**

This is a qualitative, descriptive and analytical cross-sectional study realized between April and May 2020 with the general public using an anonymous online questionnaire covering: socio-demographic data; behavior during confinement; epidemic psychological impact; Mental health status was measured using Depression, Anxiety and Stress Scale(DASS-21). Anger level was assessed by STAXI-10 items.

**Results:**

132 responses were received: 68.2% were women; the average age 32.01(±11.10); half were single; 77.3% were at higher education level; 41.7% were healthcare professionnels. Consumption increased by 26.5% in coffee, 8.3% tobacco and 1.5% alcohol. 33.3% of participants increased their religious practice. 56.1% experienced sleep disorder mostly women(p<0.05). 91.7% followed COVID19 evolution through media essentially Facebook. Partners relationship was deteriorated in1.7% and improved in 25%. Child abuse increased by 13.7%. During confinement: 15.9%depression (0.8%severe depression), 12.9%anxiety (0.8%severe anxiety), 5.3% stress and 27.3%feelings of anger. Depression, anxiety and anger were related to younger age(p<0.05). Stress wasn’t age related. Depression was observed in participants without children (p<0.05). No association founded between psychological impact and gender, profession and civil status.

**Conclusions:**

The COVID19 pandemic and the containment had consequences on individuals behavior and mental health. A psychological listening unit was launched during the period of confinement to overcome psychological impact.

